# Coronavirus 2019 (COVID-19) outbreak and geropsychiatric care for older adults: a view from Turkey

**DOI:** 10.1017/S1041610220001167

**Published:** 2020-06-11

**Authors:** Mehmet Ilkin Naharci, Bilal Katipoglu, Ilker Tasci

**Affiliations:** 1 Division of Geriatrics, Gulhane Faculty of Medicine and Gulhane Training and Research Hospital, University of Health Sciences Turkey, 06010, Ankara, Turkey; 2 Department of Internal Medicine, Gulhane Faculty of Medicine and Gulhane Training and Research Hospital, University of Health Sciences Turkey, 06010, Ankara, Turkey

## Introduction

The coronavirus disease 2019 (COVID-19) outbreak in Turkey, caused by severe acute respiratory syndrome coronavirus-2, is a reflection of the global public health crisis, which was declared by the World Health Organization on March 11, 2020 (World Health Organization, 2020a). As the virus is highly contagious and can quickly spread among people, cases started to appear across the country in a short time.

All age groups in the community are at risk of becoming infected with the COVID-19. However, older adults are predisposed to have a more severe disease due to comorbid situations, including hypertension, diabetes, coronary heart disease, chronic obstructive heart disease, and renal failure, which reduce vitality and resilience (Zhou *et al.*, 2020). Age-related decline in physiological reserve and the altered innate immune system, called immunosenescence, increases the risk of severe infections in older adults (Weiskopf *et al.*, 2009). But the knowledge obtained from the previous outbreaks or flu seasons could not explain the unexpectedly higher risk of death with the increasing number of chronic diseases of aged people. While no evidence-based treatment strategy exists presently, social distancing has been the primary measure to prevent the spread of the virus. In this situation, the COVID-19 outbreak may cause some mental health issues in older individuals with no mental illness, as well as recurrence of the disease in people with psychiatric illness and stress in both themselves and relatives. In response to this process, the changes in the delivery of mental health services have to meet the needs of older adults better worldwide.

This paper focuses on Turkey’s fight against the COVID-19 pandemic, the problems in geropsychiatric care, and their solutions encountered during the period of social isolation of older adults. The precautions to be taken during the outbreak, which seems to have a dynamic process, may change rapidly. This challenge may continue until we have a coronavirus vaccine.

### Epidemiology

The first confirmed case of COVID-19 infection was reported on March 10, 2020, much later than in many European countries (Turkish Ministry of Health, 2020a; World Health Organization, 2020b). The first report of death from COVID-19 came on March 17, 2020, when the total number of cases had reached 98 (Turkish Ministry of Health, 2020a). As of May 12, 141,475 confirmed cases and 3,894 deaths from COVID-19 reported by the Turkish Ministry of Health. The preliminary estimated mortality rate is 2.75%, which is lower than the estimated global average of 6.2% (Turkish Ministry of Health, 2020b; World Health Organization, 2020c). However, these numbers are calculated among the closed cases and are subject to change dynamically as the number of discharged individuals increase.

Although the Turkish Health Minister’s daily briefings have persistently addressed the disease as disproportionately severe in older people, information on confirmed cases and deaths in different age groups was still not available to the public as of May 12, 2020.

### Prevention strategies

As early as January 2020, when there were no confirmed cases, the Turkish Ministry of Health established a coronavirus scientific advisory board to prevent transmission of the virus to the country and spread of COVID-19 infection. Similar to other countries, the Turkish Ministry of Health released various protocols to implement prevention strategies. Turkey took the most vigorous preventive measures to tackle the spread of COVID-19 infection in public and alleviate the potential impact of the epidemic (Anadolu Agency, 2020a). These measures likely contributed to the reduced number of new cases and deaths. Nevertheless, no scientific evidence is available on whether the self-isolation of older people was independently useful in preventing the spread of the disease.

### Medical intervention

On January 2020, “COVID-19 Risk Assessment,” “COVID-19 Treatment Guideline,” and “Case Report Form” for the medical management were prepared and updated based on suggestions of the coronavirus scientific advisory board (Demirbilek *et al.*, 2020). Clinicians implemented standard procedures for all confirmed and suspected cases with COVID-19 infection across the country. The Turkish Ministry of Health provided testing and health care services for coronavirus free of charge (Anadolu Agency, 2020b). Health officials have announced persistently to the public that age and additional comorbidities have never been the primary indicators of selecting or avoiding any particular medical treatment. Moreover, the use of any primary pharmacologic intervention has never come to the fore in older people despite the obvious risk of severe disease following COVID-19 infection.

### Mortality

The relatively lower number of deaths from COVID-19 infection in Turkey is increasingly under debate. The approach of early identification and hospitalization of patients at risk of severe disease has been indicated by the authorities to underlie the low rates of dying from COVID-19 infection. Nevertheless, there has been no scientific report so far to support this hypothesis.

Another explanation of the lower death rates in Turkey compared to most European countries may be the lower percentage of older people in the population. The latest figures among the Organisation for Economic Co-operation and Development (OECD) countries show that 8.6% vs. 22.7% are older adults in Turkey and Italy, respectively, while the average of the OECD is 17.2% (OECD, 2020). As health officials urged this relatively small percentage of the population to stay home even before the documentation of the first case and further restrictions were explicitly performed to them in the following days, a lower number of elderly admissions to hospitals could be maintained.

## National health system and psychogeriatric care during the COVID-19 outbreak and recommendations

The current social security system managed by the Social Security Institution (SCI) covers almost all citizens in Turkey (Republic of Turkey Social Security Institution, 2016). Following the onset of the outbreak, the national budget included the costs of screening, hospital admissions, and treatment for COVID-19 explicitly (Anadolu Agency, 2020c). Furthermore, primary, secondary, and tertiary care settings are disseminated across the country and well managed by a single. Integrating health care services to the entire population likely contributed to the mitigation of the spread and impact of the outbreak.

### Outpatient care

COVID-19 has had a significant impact on routine medical appointments for psychogeriatric care in Turkey (Psychiatric Association of Turkey, 2020). Many patients postponed outpatient psychiatric visits as they did not leave home to reduce the risk of exposure to virus. Health providers advised older adults to admit to hospitals only in case of emergency. The number of in-person visits was limited in many psychiatric clinics. Follow-up appointments were suspended and planned after the outbreak (Psychiatric Association of Turkey, 2020). However, some outpatient clinics continued to manage psychogeriatric care for patients who were in their follow-up via telephone and video (Medimagazin, 2020). But there is no legal basis for this care in Turkey. On the other hand, to provide maintenance medication, the governor’s emergency order provided access to 30-day refill of prescription medications for chronic conditions from the local pharmacies without visiting the family physicians or other outpatient settings to get the previously required code (Turkish Ministry of Health, 2020c). This order covered drugs that are under more strict prescription control.

The solution to this challenge could be telemedicine, which can be useful for outpatient clinical care of patients with chronic conditions (Punia *et al.*, 2020). Accumulating evidence supports that live-video conferencing is a helpful and safe way to manage mental health problems, and improves both psychological well-being and chronic medical conditions in older adults (Batsis *et al.*, 2019; Punia *et al.*, 2020). Incorporating telemedicine into outpatient health care may yield favorable outcomes in the long term (Batsis *et al.*, 2019). However, it is also noteworthy that many older adults face a lot of difficulties in adopting digital technologies such as lack of necessary skills and incentives, limited access due to economic obstacles, and disability. Therefore, digital technology training is required to be widespread among older adults.

### Nursing home care

There are about 400 nursing homes and long-term care facilities in Turkey housing about 30,000 people (Sahinli and Tarım, 2019). As of the day this paper was submitted, information on confirmed cases and deaths in these settings was not available to the public.

As of March 2020, a guideline developed by the Turkish Ministry of Health and targeted at reducing the impact of the outbreak on nursing homes was released and updated to reduce the risk of contamination during nursing home care (Turkish Ministry of Health, 2020d). The measures in this guideline taken against COVID-19 were as follows: infection prevention and control principles to protect residents and staff, implementing some of the practices in case of identifying suspected patients, monitoring all residents daily, testing residents every 14 days, restriction all visitors, canceling all group activities and communal dining, and 14-day shifts for staff.

On the other hand, families were concerned about the health status of residents, after banning visits to nursing homes. To reduce fear, loneliness, and coping with stress at this time, nursing homes offered residents communicate with loved ones by telephone or video calls (Anadolu Agency, 2020d).

### Home care services

There are about 500,000 entirely or mostly homebound older adults living in Turkey, who receive care and follow-up by home health professionals (Turkish Ministry of Family and Social Policies, 2019a). Home health services are given by the Ministry of Health, which was covered by SCI’s home health benefits, and SCI-certified private agencies.

The outbreak led to an increased demand for home health care, especially some non-homebound older adults who want to receive this without admission to the hospital (Anadolu Agency, 2020e). Although home care services continue for homebound older adults at this time, supporting and boosting the capacity of home health care are required to respond timely and appropriately to coming to other disasters.

### Caregiver burden

The pandemic could also raise the stress of caregivers of individuals who needs assistance with physical or cognitive limitations. The behavioral and psychological symptoms such as anxiety, panic attack, depression, and delirium could be aggravated in those vulnerable people at this time, which can be physically and emotionally exhausting and stressful to caregivers. This condition could adversely affect the mental and physical health of caregivers and increase the risk of abuse and neglect to an older adult.

With the aim of reducing this burden, the telephone intervention called “COVID-19 Psychosocial Support service” by a team of psychologists and social workers is available to support the caregivers of individuals with dementia in Turkey (Turkish Ministry of Family and Social Policies, 2019b). But there is a need for broader information across the country on this service that can help caregivers overcome encountered challenges. Past pandemics revealed that caregivers suffer significant morbidity from the care of patients with various diseases (Ho *et al.*, 2020). Lack of available information worldwide on the impact of COVID-19 on mental health and caregiver burden requires further investigation.

### Psychological and behavioral impacts on engagement with novel preventive measures

As mentioned earlier, following the announcement of the COVID-19 outbreak as a pandemic, many countries urged people to stay at home in self-quarantine until a second statement. Turkey subsequently announced restrictions for older adults with chronic illnesses to stay home (Anadolu Agency, 2020f). Nevertheless, despite massive, nationwide campaigns, many older individuals have initially failed to engage in staying at home and tried to go out for various reasons such as exercising, meeting friends, dog walking, and shopping (Daily Sabah News, 2020; Hurriyet Daily News, 2020; LabMedya Media Channel, 2020). Interestingly, a widespread perception has been young people adhered to this rule more than older adults (ABC News, 2020). What could underlie such a non-adherence with staying home that aged people showed at the cost of putting their lives at risk?

Several cognitive and psychosocial factors can cause an unwillingness to stay at home among older adults. First, aging is associated with a broad range of cognitive changes from mild cognitive impairment to dementia. Age-related decline in specific cognitive tasks such as working memory, processing speed, perception, executive function, language, and visuospatial functions is a part of the normal aging process (Harada *et al.*, 2013). In line with this, older people may have difficulty in searching and learning new methods (Frey *et al.*, 2015). Cognitive aging may also impair the ability to make intelligent decisions (Frey *et al.*, 2015). These may partly explain why many older individuals remained less responsive to action calls and campaigns in Turkey and other countries during the early period of the pandemic.

Second, mood disorders such as anxiety and depression are more common in multimorbid individuals but often subtle. Patients with anxiety/depression are more likely to show poor compliance, suggesting that accelerated decline in cognitive abilities might impair understanding, appreciation, and reasoning (Piotrowicz *et al.*, 2016). In rare instances such as the COVID-19 pandemic, mood disorders may reduce prompt compliance of older people with preventive measures at the population level. Moreover, low compliance may, in part, associate with self-neglect, which is more frequent in depression (Papaioannou *et al.*, 2012). Thus, new approaches for those with mood disorders may be necessary to engage in self-isolation during such outbreaks.

Lastly, some older people might show an unintentional self-reaction to isolation to maintain their social contacts that merely occur out of the home, such as at daycare services, senior centers, living communities, and places of worship, with the risk that this population feels lonely (Armitage and Nellums, 2020). Mainly older adults who have lower education levels have the habit of spending time by talking to each other, and walking outside may be an underlying reason for this situation. Conversely, more educated older people have a positive attitude toward technology and have a better time at home, using the Internet and social media (Chiu and Liu, 2017). Also, providing and maintaining self-isolation in older adults can be difficult in collective, life-loving societies like ours.

### How to increase engagement with isolation and social distancing among older people?

Remote treatment options via television, Internet, and mobile phone applications may potentially help adapt or overcome it (Khosravi and Ghapanchi, 2016). As such, several types of cognitive-based interventions have been found effective in older adults (Lautenschlager *et al.*, 2014). These include cognitive stimulation, cognitive training, cognitive enrichment, and cognitive rehabilitation (Lautenschlager *et al.*, 2014). Cognitive-behavioral therapy delivered by the Internet for depression and anxiety helps to reduce disease-related symptoms, functional impairment, and distress in primary care (Newby *et al.*, 2017).

Older individuals warrant particular attention in promoting an active lifestyle at home during a pandemic. Encouraging older adults to develop a daily routine, make telephone or video calls with family members and friends, engage in joyful and pleasant home activities, and maintain regular exercise during this outbreak are other interventions that may improve psychosocial function and quality of life and provide staying indoors.

## Conclusion

In Turkey, steps toward normalization in social life, also called as a new healthy life under coronavirus, have been being discussed since the beginning of May 2020. However, older adults are still advised to social distancing, which will likely be critical to the so-called “new normal life” soon. We do not know how long this social distancing and isolation period will last, but we see this outbreak will not be the last. This latest outbreak has shown the lack of adequate, human-centered management plans in the context of elderly care during a pandemic. Extraordinary measures, such as meeting physical, mental, and social needs, should include a detailed description of isolation goals and approaches that increase motivation in older adults.
